# High-performance solution-based CdS-conjugated hybrid polymer solar cells

**DOI:** 10.1039/c8ra01813h

**Published:** 2018-05-18

**Authors:** M. Imran, M. Ikram, A. Shahzadi, S. Dilpazir, H. Khan, I. Shahzadi, S. Amber Yousaf, S. Ali, J. Geng, Y. Huang

**Affiliations:** Technical Institute of Physics and Chemistry, Chinese Academy of Sciences 29 Zhongguancun East Road, Haidian District Beijing 100190 China jianxingeng@mail.ipc.ac.cn yhuang@mail.ipc.ac.cn; University of Chinese Academy of Sciences Beijing 100049 China; Solar Cell Applications Research Lab, Department of Physics, Government College University 54000 Lahore Punjab Pakistan dr.muhammadikram@gcu.edu.pk; University College of Pharmacy, University of the Punjab Lahore 54000 Pakistan; Key Laboratory of Green Process and Engineering, Institute of Process Engineering, Chinese Academy of Sciences Beijing 100190 China; Department of Physics, School of Science, University of Management and Technology Lahore 54770 Pakistan; Department of Physics, Riphah Institute of Computing and Applied Sciences (RICAS), Riphah International University 14 Ali Road Lahore Pakistan

## Abstract

In this study, hybrid BHJ – bulk heterojunction polymer solar cells were fabricated by incorporating CdS quantum dots (QDs) in a blend of P3HT (donor) and PCBM (acceptor) using dichlorobenzene and chlorobenzene as solvents. CdS QDs at various ratios were mixed in a fixed amount of the P3HT and PCBM blend. The prepared samples have been characterized by a variety of techniques such as *I*–*V* and EQE measurements, atomic force microscopy (AFM), scanning electron microscopy (SEM) and ultraviolet-visible (UV-vis) spectroscopy. The mixing of QDs in the polymer blends improved the PCE – power conversion efficiency of the solar cells under standard light conditions. The improved PCE from 2.95 to 4.41% is mostly due to the increase in the fill factor (FF) and short-circuit current (*J*_sc_) of the devices with an optimum amount of CdS in the P3HT:PCBM blend. The increase in *J*_sc_ possibly originated from the formation of a percolation network of CdS. The conjugation of QDs has increased the absorption of the active layers in the visible region. These results well matched as reported, conjugation of CdS in the perovskite active layer increased the absorption and PCE of the devices relative to those of the perovskite films. This increment in parameters is attributed to the decrease in charge recombinations that improved the performance of the doped device.

## Abstract 2

In this study, hybrid BHJ – bulk heterojunction polymer solar cells were fabricated by incorporating CdS quantum dots (QDs) in a blend of P3HT (donor) and PCBM (acceptor) using dichlorobenzene and chlorobenzene as solvents.

## Graphical abstract

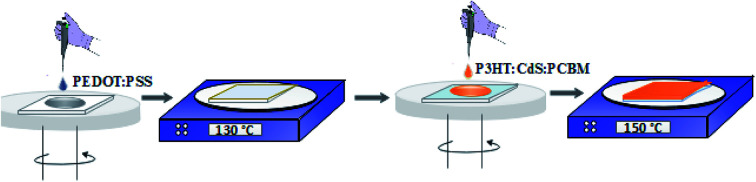

## 1. Introduction

Bulk heterojunction (BHJ) polymer solar cells (PSCs) have demonstrated great potential for assembling mechanically flexible and large-area panels through cost-effective solution processing techniques.^1–4^ Conventionally, BHJ-based devices have an active layer of the conjugated polymer P3HT – poly(3-hexylthiophene) as an electron donor-D and the organic compound PCBM – (6,6) phenyl-C61-butyric acid methyl ester as an electron acceptor-A.^5–9^ Still, solution-based PSCs have low PCE – power conversion efficiency relative to inorganic the semiconductor Si and CIGS-based solar cells.^10^ The low PCE is due to low charge mobility and short diffusion length (5–20 nm) of the conjugated polymers and organic SC blend-based active layers. The PCE of PSCs can be improved by controlling the film thickness and annealing temperature, selecting a proper buffer layer, and using D/A materials and low work function electrodes.^11–16^ Another issue with molecular semiconductors is that they have low dielectric constant; excitons rather than free electrons and holes are generated as a result of photoexcitation. Exciton dissociation occurs due to internal field difference between D and A.

To overcome above stated issues of PSCs, a recent trend is towards the addition of inorganic SCs nanostructured have turned to hybrid organic–inorganic devices. These hybrid active layers of conjugated polymers and inorganic semiconductor nanocrystals (NCs) have the benefit of high charge mobility and chemical and physical stability as compared to organic SC materials.^17^ Oh *et al.* incorporated CdS NPs (nanoparticles) in the active layer of P3HT:PCBM, and the resulting devices exhibited improved *V*_oc_ – open circuit voltage, *J*_sc_ – short-circuit current density, FF – fill factor and PCE.^18^ Yoon *et al.* investigated the performance of a ternary blend with PbS-NP and reported a 47% increase in the efficiency.^19^ Ikram *et al.* utilized TiO_2_, NiO, and CuO-NPs in P3HT:PCBM-based conventional and inverted systems to replace PCBM and P3HT, and increased PCEs of the device with an optimum doping concentration of NPs were achieved.^20–22^

To date, a variety of nanostructured PbSe, CdSe, PbS,^11,23,24^ FeS_2_,^25^ In_2_S_3_,^26^ Ag_2_S,^27^ and CdS^28–34^ have been used with polymers to enhance the performance of the devices. These small-sized dispersed NCs in the polymer active layer may increase the interface area between D and A, which, as a result, assists in charge separation for photogenerated carriers due to the direct pathway provided by the NCs. Furthermore, QDs are predominantly compatible with applications in hybrid organic BHJ devices, which also have high electron mobility, chemical stability and tunable absorption to capture solar light.^35^ Conversely, to achieve controllable formation of a bicontinuous percolation network, selection of solvents for phase separation in the active layer, a precise interface between the polymer matrix and QDs remains challenging.^36^ Therefore, the next generation of hybrid QD photovoltaics will require a strategy for controlling the phase separation, increasing the interfacial areas, and improving the optoelectronic interactions between inorganic QDs and organic polymers.

CdS NPs have emerged as promising materials for solar cell application and have been used as ETL in PSCs. They have also been employed in bulk-heterojunction perovskite solar cells and demonstrated enhanced absorption and PCE.^37^ In this study, the synthesized inorganic semiconductor CdS QDs at various concentrations (2, 4 and 6%) were incorporated into the active layer of the polymer (P3HT:PCBM) dissolved in CB and DCB (1 : 1). These various concentration-based active layers were sandwiched between a PEDOT:PSS-coated ITO cathode and a LiF/Al anode. The pure and doped films were characterized by a variety of techniques to check the surface morphology, film roughness, and optical and electrical properties of the devices.

## 2. Experimental details

### 2.1 Materials

ITO-deposited glass substrates with *R*_sh_ = 8–12 Ω □^−1^ were purchased from Delta Technologies USA. The hole transport material PEDOT:PSS – poly(3,4-polyethylene dioxythiophene polystyrene sulfonate) and highly regioregular P3HT and PCBM were obtained from Heraeus Material Technology LLC and BASF Corporation, USA, respectively. Cadmium chloride (CdCl_2_·2H_2_O) and ethylene glycol (C_2_H_6_O_2_) were of analytical reagent grade and purchased from Merck.

### 2.2 Synthesis of CdS QDs

CdS QDs were synthesized using 0.02 M of CdCl_2_·2H_2_O and an equimolar amount of C_2_H_5_NS dissolved in 120 ml of C_2_H_6_O_2_ as a solvent by the co-precipitation method.^38^ The dissolved solution was stirred on a hotplate at 100 °C for 4 h until the appearance of brown precipitates. These precipitate were filtered, washed with C_2_H_5_OH and deionized water, and finally dried at 100 °C to obtain the CdS nanopowder.

### 2.3 Device fabrication

**Fig. 1:**
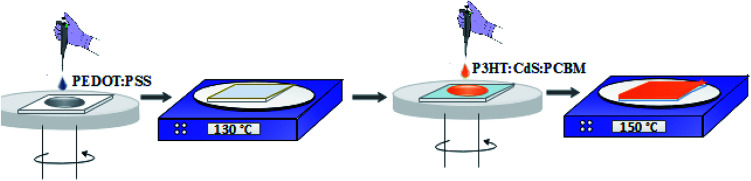
Schematic of the fabrication and annealing of spin-coated films.

The ITO surface was cleaned using an ultrasonic bath for 10 minutes each in soap, tap water, anhydrous ethanol, and acetone, rinsed with DI water, and dried under the N_2_ gas stream. Then, a thin layer of PEDOT:PSS (∼30 nm) was spin coated at 3000 rpm for one minute on the cleaned ITO (Fig. 1).

The PEDOT:PSS-deposited film was sintered on a hot plate at 130 °C for 10 minutes to reduce the roughness of the ITO surface. After this, P3HT : PCBM : CdS at various weight ratios (1 : 0.8 : 0, 1 : 0.8 : 0.2, 1 : 0.8 : 0.4 and 1 : 0.8 : 0.6) was dissolved in CB and DCB (1 : 1). These photoactive layer solutions were stirred overnight at 40 °C prior to spin casting at the top of the PEDOT:PSS layer. The spin-coated active films were annealed at 150 °C for 15 minutes in a N_2_-filled glovebox (Fig. 1). Finally, a buffer layer of LiF (0.3 nm) and aluminium contacts (100 nm) under high vacuum were deposited on the annealed films. The contact cell area of 0.2 cm^2^ through a shadow mask was maintained for all the devices.

### 2.4 Characterization

The structural analysis and particle size of the prepared CdS were confirmed using a PANalytical X'Pert PRO X-ray diffraction system ((XRD) Company Ltd. Holland) operated at 40 kV and 40 mA with CuKα radiation of *λ* = 1.54 Å; the surface morphologies and microstructures of the synthesized nanostructures and films were obtained by a field emission scanning electron microscope (FESEM JSM-5910 (accelerating voltage 20 kV)) and an atomic force microscope (Ambios, Q250), respectively. The absorption spectra were obtained by the Genesys 10S UV-vis spectrophotometer. The *J*–*V* curves were acquired using a N_2_-filled glove box *via* a solar simulator (CT 100 AAA) with the Keithley 2420 source meter under standard conditions (AM 1.5 G, 100 mW cm^−2^ irradiation intensity). The external quantum efficiency (EQE) measurements of the devices were conducted in the air by the Model QEX 10 (PV Measurement) system.

## 3. Result and discussion

**Fig. 2:**
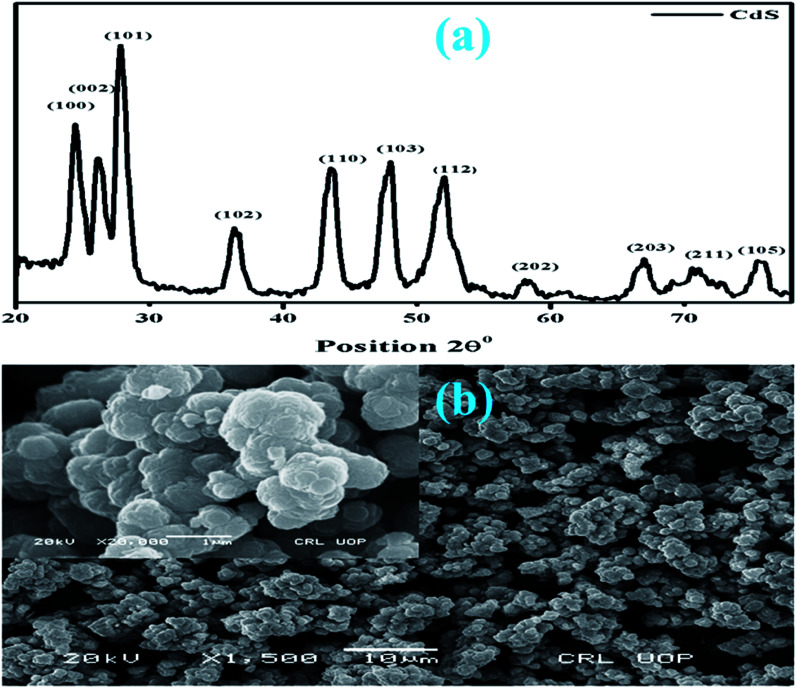
XRD pattern of the synthesized CdS nanostructures (a) and the SEM image (b).

Fig. 2(a and b) show the powder X-ray diffraction (XRD) patterns and scanning electron microscopy (SEM) images of the CdS-QDs prepared by the co-precipitation method. The observed peaks of the as-prepared QDs indicate a hexagonal close-packed (hcp) structure and well match with the JCPDS card # 01-075-1545. The calculated crystallite size of CdS is around 17.30 nm obtained using the Debye Scherrer's formula.^38^ To analyze the surface morphology of CdS-QDs, scanning electron microscopy was performed on the samples (Fig. 2b). In the figure, the formation of highly agglomerated spherical NPs for CdS was observed.

**Fig. 3:**
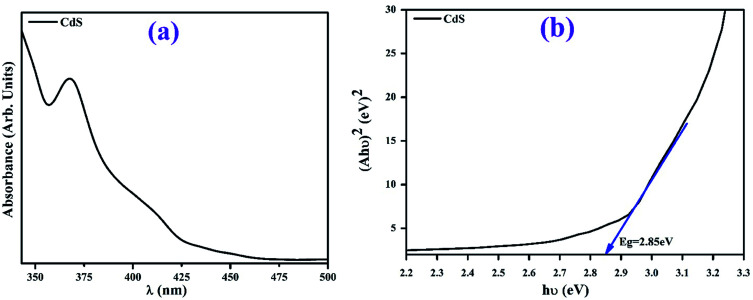
UV-vis spectrum of the synthesized CdS (a) and the measured band gap obtained by Tauc's plot (b).

Optical properties of CdS-QDs were probed by UV-vis spectroscopy, as shown in Fig. 3(a and b). Pristine CdS QDs demonstrate absorption between 325 and 460 nm in the UV and visible regions. The energy band gap measured from the absorption spectrum for CdS was around 2.85 eV,^39^ calculated using the Tauc's equation, as shown in Fig. 3b.

**Fig. 4:**
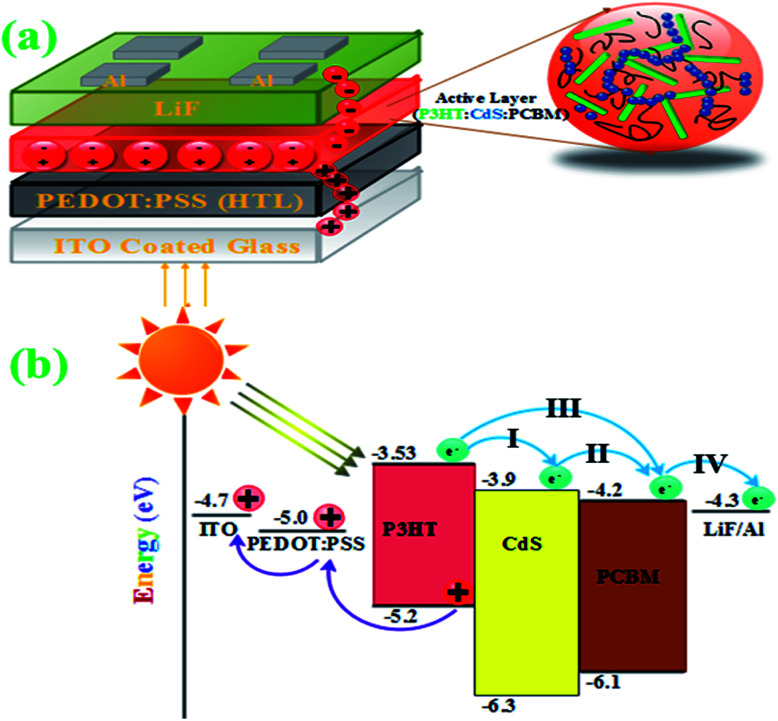
Schematic and (a) the energy scheme of the prepared devices (b).

Fig. 4(a and b) show the schematic of the conventional OPV – organic photovoltaics and energy level diagram of the components^21,40^ proposed for CdS-conjugated devices, respectively. The donor material P3HT in the active layer absorbed incident photons and generate electron–hole pair (exciton) at the interface of D and A (Fig. 4a). The generated electrons have different paths (I, II, and III) to reach the anode (LiF/Al), and the holes move towards the HTL (hole transport layer) PEDOT:PSS-coated ITO (Fig. 4b).

**Fig. 5:**
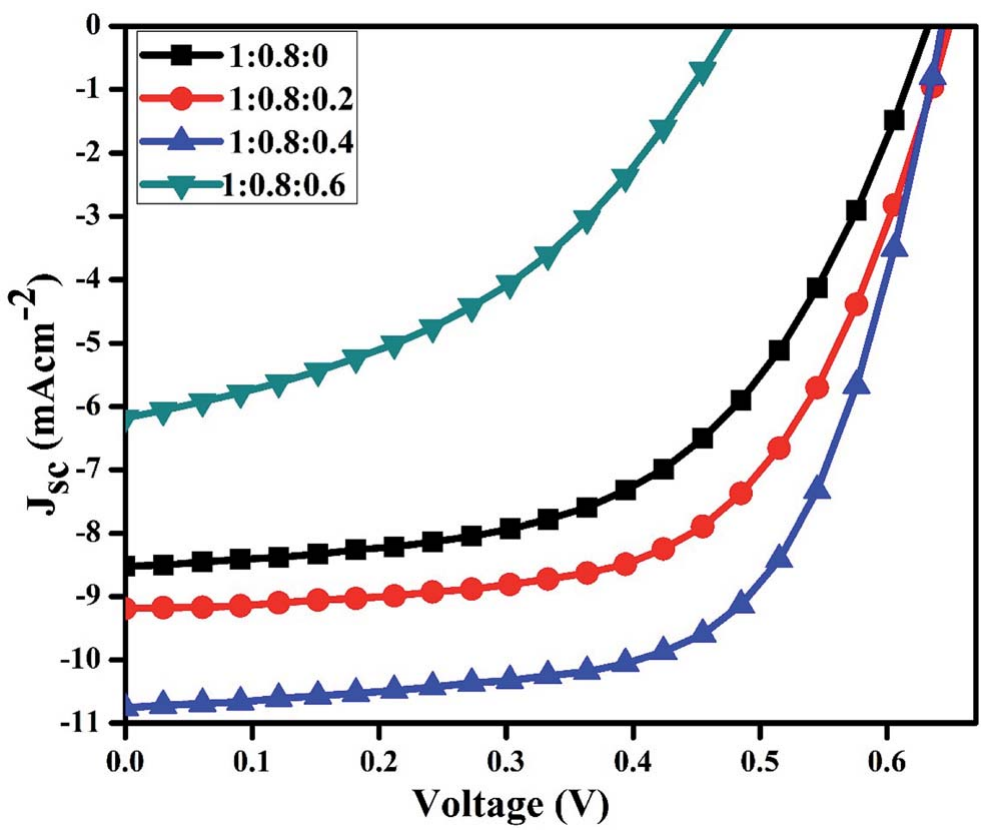
*J*–*V* curves of the binary and ternary blended hybrid organic solar cells.

To determine the best performing device, *J*–*V* characteristics of binary and ternary systems at various concentrations were measured under the light conditions of 100 mW cm^−2^ (Fig. 5).

It is clear from the figure that PCE increases when different amounts of CdS-QDs are incorporated into the P3HT:PCBM active layer. The PCE increased from 2.95 to 4.4% at the optimized concentrations of CdS in the P3HT:PCBM blend. This increase in efficiency is attributed to the increases in FF and *J*_sc_ upon doping of QDs to the active layer of the devices. Various ratios of the active layer blends were used, and the corresponding electrical parameters obtained from Fig. 5 are listed in Table 1.

**Table 1:** Device parameters obtained from the *J*–*V* curves shown in Fig. 5. All values have <4% SD – standard deviation

P3HT : PCBM : CdS	*V*_oc_ (V)	*J*_sc_ (mA cm^−2^)	FF (%)	PCE (%)
1 : 0.8 : 0	0.63	8.5	54.97	2.95 ± 0.1
1 : 0.8 : 0.2	0.65	9.2	60.18	3.66 ± 0.05
1 : 0.8 : 0.4	0.64	10.8	63.59	4.41 ± 0.03
1 : 0.8 : 0.6	0.48	6.2	41.78	1.24 ± 0.2

The increase in PCE should be ascribed to the incorporation of CdS QDs, which form a network structure and may improve the heterojunction in the active layer. A significant increase in *J*_sc_ was found upon mixing as a result of an increase in the absorption and formation of an interpenetrating network of QDs in the active layer. The increase in *J*_sc_ suggests that the presence of CdS leads to more effective charge transfer at the interface and reduces recombination losses.^37^ Upon incorporation in the polymer blend, CdS nanoparticles also act as electron acceptors due to their low energy level as compared to P3HT. The conduction band level of CdS (3.9 eV) effectively increases the difference between the LUMO of the acceptor and the HOMO of the donor.^41^ The addition of CdS caused reorganization of the energy levels between the HOMO of P3HT and the conduction band (CB) of CdS. Although it was reported that the reorganization of energy levels could result in changes in *V*_oc_ of the resulting devices,^42,43^ in this research, the *V*_oc_ kept constant (ignorable changes) at low loadings of CdS QDs into the active layer. The low efficiency of the device with the ratio 1 : 0.8 : 0.6 was attributed to agglomeration of CdS. The excess amount of QDs in the blend can damage the interpenetrating network for charge transport and lead to poor solar cell performances.

**Fig. 6:**
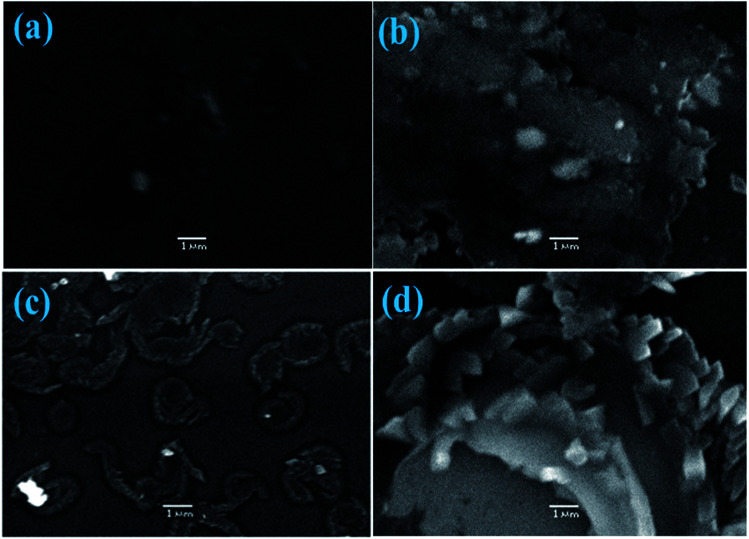
FESEM images of the undoped and doped films of P3HT:PCBM.

To verify the dispersibility and behavior of QDs in the polymer active layer, field emission scanning electron microscopy (FESEM) was conducted on the films. The FESEM micro-images of binary and CdS-doped (2, 4 and 6%) P3HT:PCBM-blended films are shown in Fig. 6(a–d). Fig. 6a depicts an image of a CdS-free film (1 : 0.8 : 0) spun from CB, and DCB seems smooth as compared to the doped films Fig. 6(b–d). With the optimized amount of CdS (1 : 0.8 : 0.4), the film shows an interpenetrating network of the P3HT : PCBM : CdS-based surface, which may facilitate electron transportation in the active layer. The excess amount of CdS-doped films (1 : 0.8 : 0.6) and complete agglomeration of QDs were found, as shown in Fig. 6d.

**Fig. 7:**
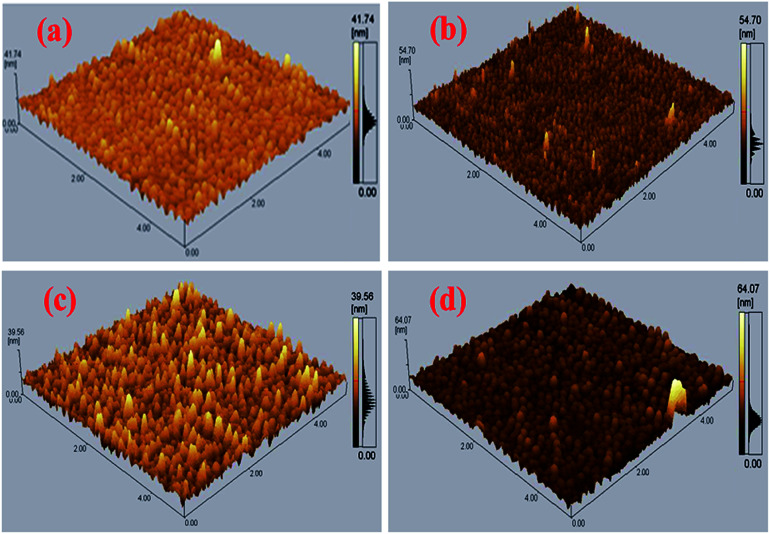
AFM images of the binary and ternary blended active layers.

To determine the active layer roughness, morphology, and agglomeration of CdS-QDs in the blend of P3HT:PCBM, AFM was conducted on the annealed films, as shown in Fig. 7. The control film P3HT:PC_61_BM (Fig. 7a) shows a smooth surface with an RMS – root mean square of 2.1 nm. After incorporating a small amount (2%) of CdS QDs (Fig. 7b), a rough surface film with a roughness value of 2.22 nm was obtained. Further addition of CdS in the blend of P3HT and PCBM-based active layer resulted in the RMS values of 2.99 and 3.66 nm corresponding to the films ratio 1 : 0.8 : 0.4 (Fig. 7c) and 1 : 0.8 : 0.6 (Fig. 7d), respectively.

**Fig. 8:**
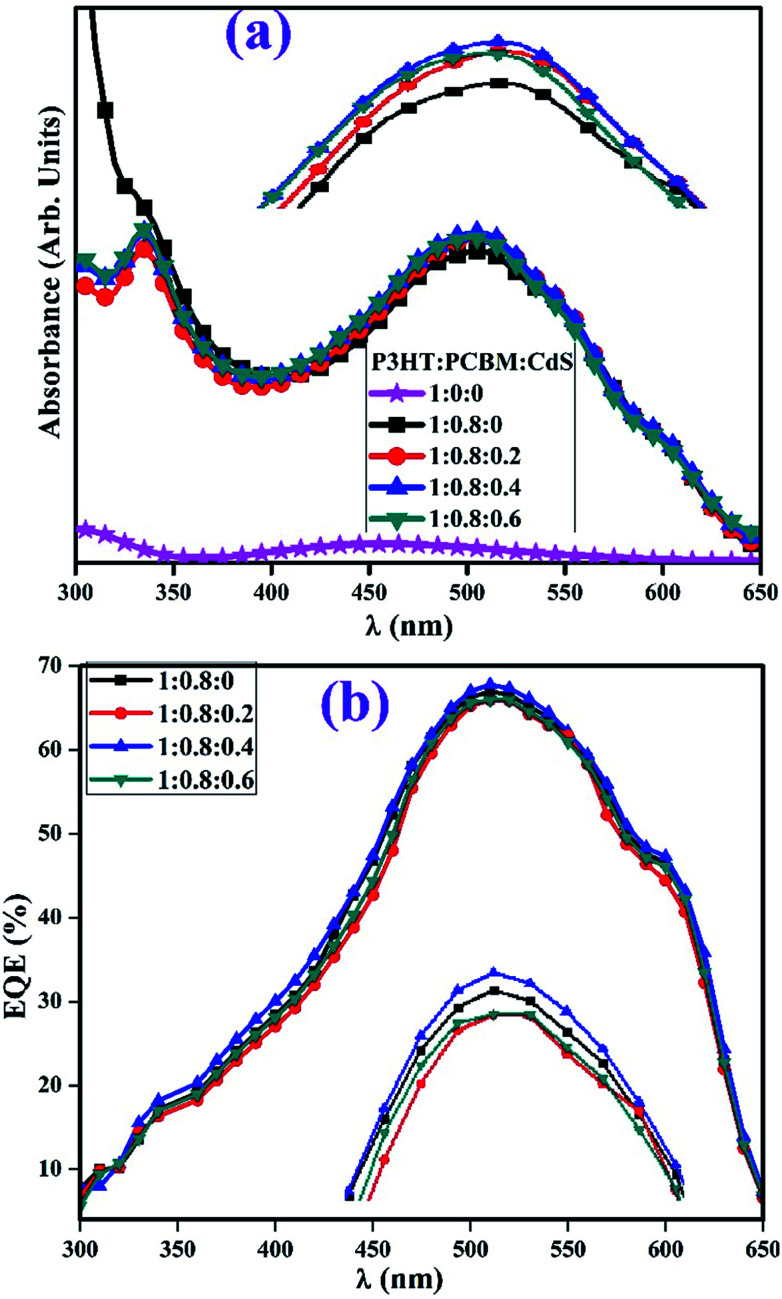
Absorption spectra of the undoped and CdS-doped films of P3HT:PCBM (a) and EQE curves of the devices (b).

The UV-vis absorption spectra and external quantum efficiency of the CdS-free and doped P3HT:PCBM films are depicted in Fig. 8(a and b), respectively. The absorption increased in the visible region with the increasing concentrations of QDs in the blend of P3HT and PCBM (inset Fig. 8a). The presence of QDs in the blend of P3HT:PCBM was observed around 335 nm in the absorption spectra as compared to the case of the control film (1 : 0.8 : 0). The increase in absorption in the visible region upon doping is attributed to film roughness, which supports light scattering in the film with an increase in the absorption and IPCE – incident photon to current efficiency.^14,20^ The absorption onset 2.85 nm of CdS NPs also contribute in absorption increment in the visible region.^41^ To measure the EQE, the binary and ternary devices are illuminated by a Xe-arc lamp with monochromatic light intensity. The intensity is measured using a calibrated Newport 818-UV Si detector, and the obtained EQE curves of P3HT : PCBM : CdS-based devices are displayed in Fig. 8b. The EQE improved with an optimum amount of CdS in the active layer relative to that of the control device. The control and doped blend-based devices indicate strong photo-responses from 300 to 650 nm, which are fully consistent with the absorption spectra (Fig. 8a) of the films.

## 4. Conclusion

In summary, hybrid organic BHJ devices were successfully prepared by incorporating various ratios of CdS QDs in the active layer of P3HT/PCBM. With the optimized amount of CdS, the ternary blend shows higher PCE as compared to the binary device *t* from 2.95 to 4.41%. The improved PCE was caused mostly by the increase in *J*_sc_ and FF with CdS-conjugated active layer of the devices. All electrical parameters and EQE of the doped devices increased with the increasing amount of CdS in the active layer of P3HT:PCBM. The absorption of the doped active layers increased in the visible region than that of the polymer active layer. The film roughness increased with doping, and excess ratios of QDs in the active layer showed agglomerations and damaged interpenetrating network, leading to reduced performance of the devices; this suggested that PCBM also acted as a surfactant material for the CdS-conjugated active layer.

## Conflicts of interest

This manuscript has no conflict of interest.

## Supplementary Material
